# Pro-environmental behavioral intention in university energy-saving contexts: extending the Value–Belief–Norm framework with institutional support

**DOI:** 10.3389/fpsyg.2026.1827476

**Published:** 2026-05-22

**Authors:** Xiaolin Zhang, Songyu Jiang, Jirawan Deeprasert

**Affiliations:** Rattanakosin International College of Creative Entrepreneurship, Rajamangala University of Technology Rattanakosin, Nakhon Pathom, Thailand

**Keywords:** pro-environment, social support, sustainability-oriented education, sustainable development, university leadership, Value-Belief-Norm

## Abstract

**Objective:**

With increasing energy demands across the globe and increased focus in sustainable development agenda, higher education institutions (HEIs) are relevant in developing energy-saving cognitions and attitudes of students.

**Purpose:**

This paper will examine how university leadership, sustainability-oriented education, environmental values, environmental beliefs, and personal norms influence and affect the pro-environmental behavioral intention (PEBI) of HEIs students.

**Methods:**

A group of 864 students of 81 HEIs located in the Shanxi Province took part in a survey and were recruited through a multi-stage stratified sampling design. The data analysis was done using structural equation modeling (SEM).

**Results:**

First, sustainability-oriented education exhibits effective and multidimensional impacts which directly and indirectly impact the environmental values, environmental beliefs, personal norms, and behavioral intentions. Contrastingly, the university leadership (UL) has no direct impact on PEBI and does not trigger the personal norms in the students. Second, the Value–Belief–Norm (VBN) sequence was supported, and personal norms emerged as the strongest direct predictor of PEBI. However, environmental values and environmental beliefs also retained significant direct effects on PEBI. Third, sustainability-oriented education translates into PEBI through significant indirect pathways via environmental values and environmental beliefs, whereas the corresponding indirect effects of university leadership through these psychological mediators are weak or non-significant. Fourth, the integrated model demonstrates strong explanatory power, but this explanatory power is primarily driven by education and the VBN psychological chain rather than by university leadership.

**Implications:**

The research reveals that effective HEIs interventions should combine direct, knowledge-based educational strategies with leadership actions designed to support and enable student-driven change, rather than attempting to directly command behavioral change through institutional authority.

## 1 Introduction

The global process of urbanization, digitalization, and industrial transformation has resulted in the sudden high rate of energy demand, which imposes the greatest pressure on energy systems and the increase in sustainability issues in various regions (Zhang, 2025). The world is still increasing its demand on electricity at a rate of about 2.5 percent annually as more and more buildings are constructed, digital infrastructure is developed, and the growing energy requirements of homes are met (Castro et al., 2024). It is against this background that higher education has become a prime stage of promoting the world sustainable development agenda. The United Nations Sustainable Development Goals (SDGs) with SDG 7 (Affordable and Clean Energy) and SDG 12 (Responsible Consumption and Production) specifically invite universities not only to trim their operation footprint but also to raise environmentally friendly citizens able to contribute to a society-wide change toward energy efficiency (Küfeoglu, 2022; Olabi et al., 2023).

The Higher Education Institutions (HEIs) are organizations in the public sector which are energy-consuming. Their elaborate structures, such as laboratories, educational institutions, libraries, student hostels demand constant power supply to promote lighting, heating, air conditioning, and online forms of learning (Laporte and Cansino, 2024). Housing structures comprise close 80 percent of energy usage inside a university with the consumption behavior of students and their online lifestyles playing a significant role in total energy consumption (Gao et al., 2024). The use of electricity on the campus in China is an urgency that is facilitated by the sustainability objectives that the country is aiming at. By 2023, the electricity demand in the country had reached 8.7 trillion kWh, and the figure is steadily increasing with the growth of digitalization and the rise in living standards (He et al., 2025). In Chinese universities, electricity consumption is between 20 and 40 percent more intensive than in other institutions of higher education due to the high occupancy of dorms and the length of time that trainees use personal gadgets and the high use of air conditioning and heating (Zhou et al., 2022).

Although the importance of sustainability is generally recognized by students, there remains a large intention-action gap in their energy use consumer habits (Portus et al., 2024). The surveys conducted with a large sample of Chinese university students show that more than 60% of them do not unplug inactive electrical appliances, almost 70% use air conditioner or heating much more often, and less than 30% engage in energy-saving behaviors (Li et al., 2024). The above phenomena imply that information campaigns in and of themselves do not suffice to induce behavioral change, an in-depth investigation of the underlying psychological and institutional determinants affecting the energy-saving behavior in students is necessary. Hence, the research objectives are:

RO1: To investigate the direct relationship between university leadership and sustainability-oriented education as the external institutional support and the pro-environmental behavioral intention of students.

RO2: To confirm a direct relationship between environmental values and beliefs, and individual norms, as internal drivers in the psychological perception, and pro-environmental behavior intentions among students.

RO3: To identify the mediating effect of the environmental values, the environmental beliefs, and the personal norms in the connection between the institutional support and the behavioral intentions, and therefore to discover the internal transmission mechanisms.

## 2 Literature review

### 2.1 Theoretical framework

Value-Belief-Norm (VBN) theory is one of the most influential frameworks for explaining pro-environmental behavior (Stern et al., 1999). It conceptualizes pro-environmental action as a causal sequence in which environmental values shape environmental beliefs, beliefs activate personal norms, and personal norms motivate behavior. Traditional applications of VBN have been especially effective in explaining the internal moral-cognitive mechanisms underlying pro-environmental intention. However, in organized settings such as higher education institutions, these internal psychological processes do not emerge in a social vacuum and may be shaped by broader institutional conditions. The most recently published studies combined VBN with Theory of Planned Behavior (Syed et al., 2024; Zhang, 2025), and were able to predict complex behaviors, such as sustainable consumption and waste management. Gradually, growing numbers of researchers have expanded the application of VBN to a variety of populations and situations, such as the eco-friendly decision of tourists or involvement of students in campus sustainability (Pan and Zhou, 2024; Serio et al., 2026). The discussion of factors that are antecedent to the VBN chain is a major trend that is evident in the literature in recent times. Sun et al. (2023) also discovered that VBN + TPB offered a more detailed picture of student behaviors, whereas Kumar and Nayak (2022) have confirmed the cross-cultural applicability of the theory, indicating its cross-cultural generalizability. The present study uses VBN as the core psychological mechanism to explain how students' pro-environmental intentions are formed through the sequence of values, beliefs, and personal norms, while Social Support Theory is used to specify the institutional support conditions that precede and activate this psychological process. In this study, institutional factors are not introduced as substitutes for the VBN sequence, but as upstream contextual conditions that help explain how the Value-Belief-Norm process is activated and strengthened in the university energy-saving context. In other words, the present framework extends the traditional VBN model by incorporating institutional support as a distal layer of influence that shapes students' environmental meaning systems before these are internalized as beliefs, moral obligation, and behavioral intention. This integration allows the model to retain the explanatory strength of VBN at the psychological level while better accounting for the organizational conditions under which students' pro-environmental orientations are formed.

The social support theory is a classic example of how one can use the support of their social network (e.g., emotional, informational, instrumental) to come out of the stress period and reach the goal (House, 1987). This theory has in recent years been applied to the organizational and community context to offer a description of pro-social and pro-environmental actions. Qi et al. (2025) examined the role played by institutional support and sustainability-oriented education in influencing pro-environmental behavior by students and defined them as vital enabling factors. Based on the same observation, Nieminen et al. (2025) discovered that there is a mediating effect of social support between eco-anxiety and pro-environmental action, which implies that the supportive environment plays a key role in turning concern into behavior. This study extends this logic by defining university leadership and sustainability-oriented education as forms of institutional social support. University leadership provides directional, emotional, and instrumental support by setting a vision and providing resources, while sustainability-oriented education offers critical informational support (Aggarwal and Agarwala, 2025). These two institutional factors were selected because they represent two salient and complementary forms of support in higher education settings. University leadership reflects the strategic, symbolic, and resource-related dimension of institutional support, whereas sustainability-oriented education reflects the informational, interpretive, and practice-oriented dimension through which students repeatedly encounter and internalize sustainability-related knowledge and expectations. By integrating this theory, we position institutional factors not as external, disconnected variables, but as foundational supports that initiate and strengthen the VBN psychological process.

In the present study, social support theory is used as a contextual framework rather than as a full interpersonal support measurement model. In higher education settings, support is often institutionalized rather than purely interpersonal. Accordingly, this study captures institutional support through two context-relevant forms: university leadership and sustainability-oriented education. This conceptualization helps explain how institutional context enters the VBN framework not as an alternative causal chain, but as a distal support structure that shapes the emergence and strength of the internal Value-Belief-Norm process. These two institutional variables represent different support functions. University leadership primarily reflects institutional and instrumental support through policy direction, strategic commitment, and resource allocation, while sustainability-oriented education primarily reflects informational support by providing students with environmental knowledge, interpretive guidance, and practical energy-saving strategies (Flori et al., 2025). This distinction helps clarify why the two forms of institutional support may show different strengths across the psychological pathway. This conceptualization also strengthens the integration with Value–Belief–Norm (VBN) theory. Social Support Theory explains why institutional contexts matter by identifying the support conditions that shape students' value and belief formation (Zhang and Qian, 2024), whereas VBN theory explains how those external supports are internalized through environmental values, environmental beliefs, and personal norms to influence pro-environmental behavioral intention (Gao et al., 2024). In this way, institutional support is specified as a distal antecedent, while the VBN sequence represents the internal psychological transmission mechanism.

### 2.2 Hypothesis development

Social Support Theory is used to conceptualize university leadership and sustainability-oriented education as institutional support conditions (Iqbal and Piwowar-Sulej, 2022), whereas VBN theory is used to explain how these support conditions are translated into pro-environmental behavioral intention through environmental values, environmental beliefs, and personal norms (Batool et al., 2023). This structure extends the traditional VBN perspective by locating institutional support as an upstream contextual layer that shapes the development of the internal psychological pathway rather than treating pro-environmental intention as arising solely from individual dispositions. This structure allows the model to distinguish between upstream institutional influence and downstream psychological transmission, and it also provides a basis for testing why different forms of institutional support may produce different behavioral effects. University leadership is conceptualized as students' perceived sustainability-oriented university leadership, reflected in the extent to which university and departmental leaders communicate sustainability priorities, provide visible encouragement, and create a supportive campus climate for energy conservation (Sarwar et al., 2025c). This construct is intentionally defined at the student-perception level, because students are more likely to experience leadership through proximal and observable cues (e.g., reminders, communication, and support practices) than through direct access to high-level strategic decision making. Green University Initiatives (GUIs) have been demonstrated to positively influence students' environmental attitudes, which in turn strengthens their pro-environmental behaviors (Bimantoro and Ghazali, 2025). Furthermore, incorporating environmental and sustainability-oriented education into curricula and extracurricular activities, such as joining campus environmental groups, has been found to substantially increase students' engagement in environmental protection (Handayani and Widodo, 2024). University leadership is also critical for mediating the effects of green human resource management practices on students' PEB, indicating that institutional support can enhance the effectiveness of these practices (Al-Sabi et al., 2024). Moreover, institutional support, as a form of leadership, has been proven to mediate the relationship between sustainability competencies and environmental awareness, ultimately enhancing students' behavioral intentions toward sustainability (Arnejo et al., 2025). Hence, we posit:

H1a: University leadership has a positive effect on students' pro-environmental behavioral intention (PEBI).

H1b: University leadership has a positive effect on environmental values.

H1c: University leadership has a positive effect on environmental beliefs.

H1d: University leadership has a positive effect on personal norms.

Sustainability-oriented education is defined as the extent to which a university provides sustainability- and energy-saving-related informational support through curriculum, instruction, and campus programs, including knowledge, skills, and practice opportunities for students (Nousheen et al., 2020). The improvement of environmental citizenship of undergraduates due to sustainability-oriented curricula is a promising fact of direct correlation between educational measures and pro-environmental performance (Telešiene et al., 2021). Regarding this, the development of sustainability competencies, achieved by the use of generic teaching strategies, was directly linked to the student's perceptions of new paradigms of the environment and their pro-environmental attitudes (Hyytinen et al., 2023). In addition, school activities in the form of learning and extracurricular activities discussed the idea of sustainability in classrooms and attending campus environmental organizations, positively influencing the pro-environmental values and behavior among students (Papavasileiou et al., 2025). Increased intensity of green university initiatives is associated with stronger environmental attitudes among students, which in turn positively predict their pro-environmental behaviors (Bimantoro and Ghazali, 2025; Sarwar et al., 2025a). A study conducted at Cebu Institute of Technology has revealed the role of institutional support in balancing the relationship between the sustainability competencies and environmental awareness and finally improving the behavioral intentions of students toward sustainability (Arnejo et al., 2025). Also, the sustainability-oriented learning programs enhanced their environmental attitudes and intentions strongly to do so that the impact of their ecological footprints was minimized (Khazen et al., 2025). Hence, we posit:

H2a: There exists a positive influence of sustainability-oriented education on the environmental values.

H2b: There is a positive influence of Sustainability-Oriented Education on the beliefs about the environment.

H2c: Sustainability-oriented education impacts on personal norms positively.

H2d: Sustainability-oriented education positively influences student's pro-environmental behavioral intention (PEBI).

Environmental values refer to individuals long term biospheric and altruistic orientations of values that place environmental protection first and forms the distal motivational foundation in activating beliefs, personal norms, and pro environmental intentions (Aviste and Niemiec, 2023). The manifestation of environmental values largely depends on the new ecological paradigm and environmental responsibility as mediating variables, which suggests the complexity of interplay between variables that may increase PEBI with the help of educational interventions (Yang et al., 2024). In the same way, PEBI was positively linked to environmental values among the women in Rafsanjan, Iran, and education level further reinforced this association, indicating that educational interventions can be effectively used to foreigner environmental values and practices (Eslami et al., 2024). The PEBI of young people can be predicted through environmental values and mediated by risk perception and moral anger, which emphasizes the emotional and cognitive aspects, which can be used to encourage pro-environmental behaviors (Li et al., 2022). The influence of personal norms on the relationship between values and PEB is mediated by environmental values and problem awareness, as personal norms play a significant role in translating values into action (Sarwar et al., 2025b). Environmental values and attitudes mediate the influence of environmental consciousness of employees on voluntary PEBI at the work place, suggesting organizational solutions to increase environmental involvement (Aviste and Niemiec, 2023). The environmental values mediate the association between some recreational activity to PEBI that indicates that natural experience might foster values that lead to environmental behaviors (Zhang, 2025). PEBI is positively correlated with connection with nature and biosphere values, and the latter mediates this correlation, which supports the usefulness of intrinsic connection with nature in creating environmental action (Pereira and Forster, 2015). Hence, we assume:

H3: There is a positive influence of environmental values on the pro-environmental behavioral intention (PEBI).

The cognitive linkage between the abstract values and the concrete norms is captured in environmental beliefs which include the knowledge of consequences and assigning responsibility and are of vital importance in how behavioral intentions are formed (Yang et al., 2024). Ecological beliefs are indirectly affected by environmental factors and their influence on ecological behaviors has positive impacts which show that the mediation relationship between environmental factors and behaviors are affected by ecological beliefs (Li et al., 2021). The result is in line with studies that showed a significant positive correlation between environmental beliefs and green purchasing behavior and the mediating effects of these beliefs on the relationship between personal norms and behaviors (Ogiemwonyi et al., 2023). On the same note, Gawshinde et al. (2025) established that green purchasing intentions are directly affected by environmental beliefs with the moderating effect being environmental concerns. Beliefs about the environment directly and indirectly have a serious influence on eco-tourism attitudes via destination image (Yang et al., 2024). The environmental beliefs are also major predictors of personal norm, which influences recycling behaviors at a workplace (Mouro and Duarte, 2021). Hence, we propose:

H4: There is a positive impact of environmental beliefs on pro-environmental behavioral intention (PEBI).

Personal norms are described as the internalized sense of moral responsibility to do a particular thing and have been found to have the huge effect on pro-environmental actions by driving the behavioral intentions (Yang et al., 2024). Personal norms were identified to positively affect the energy-saving intentions in the most significant way, and the willingness of residents to participate in energy-saving behaviors had significant effects in the context of household energy-saving behaviors in China (Liu et al., 2025). Likewise, regarding civil servants in Taiwan, personal norms were found to be one of the most important predictors of pro-environmental behaviors and hence they will influence environmental behaviors (Fang et al., 2019). Moreover, a study of the energy-saving behavior of university students, green purchasing intentions showed that personal norms were a significant mediator of the problem of relationship between normative information and behavioral intentions, as well as indicated that they played a crucial role in the development of pro-environmental intentions (Ling and Ling, 2025; Liu et al., 2025). Moreover, when examining the pro-environmental actions of Gen Z in the tourism setting, the study revealed that the individual norm has been identified as the key predictor of sustainable transportation decisions, showing that it affects the decision-making process of diverse groups of citizens (Marino et al., 2023). Hence, we propose:

H5: Personal norms have positive effect on pro-environmental behavioral intention (PEBI).

According to the Value-Belief- Norm (VBN) theory, the cause-effect process holds that the environmental values cause environmental beliefs, which then trigger personal norms, which provokes behavioral intentions (Soemantri et al., 2025). The positive influence of environmental values has been identified on PEBI, and mediating effects are observed with the help of the new ecological paradigm and environmental responsibility that combined in creating a chain mediation effect on PEBI (Yang et al., 2024). PEBI is also impacted by university leadership and green organizational culture, since it provides an environment where sustainability is appreciated and leadership has the capability to improve PEBI through encouraging a green work environment, environmental passion that are in harmony and partially mediate the relationship between leadership efforts and PEBI (Omarova and Jo, 2022). Moreover, genuine leadership has been directly associated with the intention to be sustainable in behavior with the condition of environmental values acting as a boundary variable to reinforce the relationship between employee engagement and sustainable behavior intentions (Ahsan et al., 2024). Environmental values mediated the effect of moral norms on the intention to reuse in the framework of the green university initiatives, emphasizing the ability to contribute to the development of sustainable behavior. In addition, the environmental knowledge and behaviors are mediated by environmental attitudes and the intellectual capacity, highlighting the significance of full-fledged environmental education programs, incorporating values (Pitaloka and Kardoyo, 2024). Hence, we posit:

H6: Environmental values have positive effect on environmental beliefs.

H7: Environmental beliefs have positive effect on personal norms.

Leadership of the university which encourages sustainability should first shape what is considered significant by the students via setting an agenda on green issues (Drissi et al., 2025), therefore, allowing the issue of environmental concerns to be more salient and students to subconsciously buy into the values articulated by authorities, as is the case in the principles of the social learning theory whereby individuals internalize the values as developed by the authority figures (Aggarwal and Agarwala, 2025). On the same note, Sustainability-Oriented Education directly conveys the knowledge on environmental issues and remedies, enhancing the environmental value systems of students as the basic sources of environmental pro-behavior (Nurkhin et al., 2025). These structural supportive processes operate on the basis of environmental values in order to manipulate the behavior intentions and down-running psychological variables like environmental beliefs. The mediating role of environmental values among different institutional and personal factors on pro-environmental behaviors has been demonstrated, which indicates that the institutions can effectively advance PEBI through connecting the environmental values to institutional culture and curricula, and thus improvement of the overall environmental welfare in the higher educational institutions (Pitaloka and Kardoyo, 2024). Hence, the following hypotheses:

H8a: Environmental values mediate the relationship between university leadership and pro-environmental behavioral intention (PEBI).

H8b: Environmental values mediate the relationship between sustainability-oriented education and pro-environmental behavioral intention (PEBI).

H8c: Environmental values mediate the relationship between university leadership and environmental beliefs.

H8d: Environmental values mediate the relationship between sustainability-oriented education and environmental beliefs.

The environmental beliefs are influenced by the support of the institutions, and personal values and are important cognitive interfaces converting the abstract values and institutional cues into specific behavioral intentions and moral duties (de Costa et al., 2025). The impact of sustainability-oriented education (ESD) on pro-environmental behaviors is mediated by environmental attitudes (a type of environmental beliefs), which receive the strongest portion of ESD effects, and this aspect is of critical importance to developing the pro-environmental behaviors in students (Bala et al., 2023). Equally, the environmental beliefs also have been defined as an important mediating factor in the correlation between personal norms and green purchasing behaviors, which makes them crucial in determining sustainable consumption practices (Ssenoga et al., 2025). Moreover, sustainability-oriented education programs were apprised to significantly improve environmental attitudes and intention to act, and these are directly associated with behavioral change also giving evidence to the mediating role of environmental beliefs (Khazen et al., 2025). Personal norms which are closely correlated with environmental beliefs also mediate the association between consciousness of the consequences and sustainable behaviors and it is further highlighted that environmental beliefs mediate between sustainability-oriented education (Yang et al., 2024). The mediation plays a crucial role in developing powerful educational programs resulting in the establishment of the long-term ecological responsibility and sustainable practice by the students. Hence, it is anticipated that the influence of institutional support and the even more distant psychological variables on behavioral intentions will be linked through the influence of environmental beliefs. Hence, this study assumes:

H9a: Environmental beliefs mediate the relationship between university leadership and pro-environmental behavioral intention (PEBI).

H9b: Environmental beliefs mediate the relationship between sustainability-oriented education and pro-environmental behavioral intention (PEBI).

H9c: Environmental beliefs mediate the relationship between university leadership and personal norms.

H9d: Environmental beliefs mediate the relationship between sustainability-oriented education and personal norms.

H9e: Environmental beliefs mediate the relationship between environmental values and personal norms.

A personal norms reflect an internal moral duty to behave in a pro-environmentally-oriented manner, are important in shifting leadership values to specific behaviors (Silvi and Padilla, 2021). Considering the context of the Ugandan universities, it was observed that environmental beliefs mediated the connection among the personal norms and green purchasing behaviors, which demonstrates the relevance of the change in behavior based on beliefs in sustainable consumption (Ssenoga et al., 2025). On that note, in Australia, the impact of personal environmental norms on environmentally conscious behaviors was identified to be stronger than the effect of climate change beliefs, and indicated that personal norms may serve to intensify the effect of environmental beliefs on behaviors (Perera et al., 2022). Personal norms are entirely mediating the connection between the alleged pro-environmental organization environment and the pro-environmental behaviors at the workplace, which is crucial to the establishment of the sustainable practice in the work environment (Mouro and Duarte, 2021). Besides, when considering the impact of environmental beliefs and issues on materialism concepts in consumer enactment, the role of personal norms is positive, meaning that they can help in addressing materialistic inclinations that interfere with green purchasing (Jhawar et al., 2023). The Value-Belief-Norm theory is also capable of supporting this mediating impact since personal norm has been observed to mediate the relationship that exists between environmental beliefs and sustainable behaviors like water use among pre-service teachers (Arslan and Durmuş, 2025). Besides, personal norms have full mediation of the impact of ecological worldviews in the context of child pro-environmental behaviors, also exemplifying the efforts to shape pro-environmental behaviors with the critical impact of personal norms (Wu, 2018). Hence, the research proposes:

H10a: Personal norms mediate the relationship between university leadership and pro-environmental behavioral intention (PEBI).

H10b: Personal norms mediate the relationship between sustainability-oriented education and pro-environmental behavioral intention (PEBI).

H10c: Personal norms mediate the relationship between environmental beliefs and pro-environmental behavioral intention (PEBI).

## 3 Method

### 3.1 Data collection and sample

This study targeted full-time undergraduate students enrolled in higher education institutions (HEIs) in Shanxi Province, China. To improve sample coverage and comparability across institutional and student subgroups, the study adopted a multi-stage stratified sampling design. The sampling strategy was structured to capture variation in institutional context while maintaining proportional representation of students across academic years. At the institutional level, this design followed a stratified sampling framework, whereas student recruitment within campuses was conducted through on-site invitation rather than pure simple random sampling.

At the institutional level, Shanxi Province was first divided into four geographic-economic strata (North, Central, South, and Southeast) for sampling purposes. Within each regional stratum, institutions were further organized to ensure coverage across key institutional characteristics, including university type (public vs. private) and broad academic orientation (medicine, natural sciences, and social sciences). Based on this stratified framework, the research team implemented institution-level sampling and field access across 81 HEIs, with the aim of ensuring broad coverage of the major regional and institutional categories in Shanxi rather than relying on a small number of campuses.

A total of 1,000 questionnaires were distributed, of which 864 valid responses were retained for analysis, yielding a valid response rate of 86.4%. Eligible participants were full-time undergraduate students who were approached by trained research assistants in public campus spaces across the sampled institutions and invited to participate voluntarily in the survey. After being informed of the academic purpose of the study and the voluntary nature of participation, students who agreed completed the questionnaire on site. Among the valid respondents (*n* = 864), 45.0% (*n* = 389) were male and 55.0% (*n* = 475) were female. The academic-year distribution was broadly balanced: freshmen (27.0%), sophomores (22.5%), juniors (25.5%), and seniors (25.1%). In terms of academic discipline, the sample included students from medicine (31.7%), natural sciences (34.7%), and social sciences (33.6%). These distributions indicate broad coverage across key demographic and academic subgroups and support subgroup-based comparisons in subsequent analyses.

It should be noted that, although the study used a stratified sampling framework at the institutional level and proportionate recruitment targets across major student subgroups, the on-site invitation of students in public campus spaces may still introduce self-selection bias (e.g., students who were more available or more willing to participate at the time of data collection). To mitigate this risk, the study used multi-campus coverage, multiple field locations within campuses, proportionate recruitment targets by academic year, and on-site completion procedures. Nevertheless, this potential source of bias is acknowledged as a limitation when interpreting the generalizability of the findings.

### 3.2 Measurement

All constructs were measured using a seven-point Likert scale ranging from 1 (“Strongly Disagree”) to 7 (“Strongly Agree”). The main text provides a brief description of the operationalization of each construct, including item sources, number of items, and representative example items, while the full final wording of all measurement items is provided in Appendix 1. All items were adapted from established scales and contextually reworded where necessary to fit the Chinese university energy-saving setting, particularly by aligning the wording with campus-based energy use, sustainability education, and student daily practices.

The constructs were quantified as follows: University Leadership (UL) was measured using five items adapted from Aggarwal and Agarwala (2025) to capture students' perceived sustainability-oriented university leadership in the university context. The items focus on leadership cues that are directly observable to students, such as leadership communication, reminders, and support for energy-saving practices (e.g., “My department head usually reminds us of the importance of energy saving”). In this study, such items are used to operationalize leadership at the student-experience level, where departmental leadership behaviors function as visible carriers of broader university leadership intent. Sustainability-oriented education was assessed using five items of Nousheen et al. (2020), and it considered the level of sustainability and energy-saving education that students obtained at their universities (e.g., “My university provides a course or a lecture connected with environmental protection or sustainable development”). The scale for measuring Environmental Values (EV) had four items constructed on the model by Yang et al. (2024), with biospheric and altruistic values (e.g., “I believe that preservation of natural environment and extinction of the species is extremely important”). Environmental Beliefs (EB) were assessed using five items that are synthesis of the New Ecological Paradigm (NEP) scale, and the research on the Awareness of Consequences (AC) and Ascription of Responsibility (AR) (e.g., “I believe my personal energy saving behavior helps reduce the energy crisis”) (Yang et al., 2024). Personal Norms (PN) was also assessed using four items, which were based on Yang et al. (2024), and evaluated the personal moral obligation toward saving energy among students (e.g., “I would feel guilty if I have not turned off the lights when leaving a room”). The Pro-environmental Behavioral Intention (PEBI) was measured using five items as indicated by Lee and Tanusia (2016), which covers the willingness of the student to perform specific energy-saving activities in future (e.g., “I am willing to actively use the air conditioning less in the future”).

### 3.3 Pre-test

A pilot test was conducted before the main survey using 50 full-time undergraduate students from one university in Shanxi Province to assess item clarity, contextual appropriateness, and preliminary reliability. All measurement items were translated into Chinese and then back-translated to improve semantic consistency with the original scales. In addition, minor wording adjustments were made to ensure that the items were appropriate for the Chinese university energy-saving context. The pilot results showed acceptable internal consistency across all constructs, with Cronbach's α values ranging from 0.79 to 0.88. Respondents also indicated that the questionnaire wording was clear and easy to understand. Based on the pilot feedback, only minor revisions were made to improve readability, and no substantive changes were made to the measurement structure before the main survey.

### 3.4 Data analysis

The data was analyzed on SPSS 26.0 and AMOS 24.0. The analysis was done in few steps. First, SPSS was used to collect the descriptive statistical analysis to analyze the demographic properties of the sampled and calculate the mean, standard deviation, skewness, and kurtosis of the primary variables to be in the position to appreciate the simple data distribution. Second, reliability and validity of the measurement model was determined. Cronbach alpha and Composite Reliability (CR) were used in assessing reliability. Confirmatory Factor Analysis (CFA) was used as the measure of validity. Convergent validity was determined by analyzing the factor loadings and the Average Variance Extracted (AVE), and discriminant validity was evaluated based on the Fornell-Larcker criterion which, taking the square root of AVE of each construct and comparing it with the correlation of the construct with other constructs was used to determine the validity. Lastly, Structural Equation Modeling (SEM) was taken to the test on the research hypotheses after a satisfactory measurement model was confirmed. Various fit indices (Chi^2^/df, CFI, TLI, RMSEA, SRMR) were used to assess the overall fit of the structural model. The statistical importance of the path coefficients between the direct and indirect (mediation) effects were investigated by the bootstrapping technique with resamples of 5,000 to find out whether the hypotheses were accepted. Given the relatively low variance observed in some constructs (particularly EB and PN), bootstrapping with 5,000 resamples was also used to improve the robustness of significance testing and confidence-interval estimation for the structural paths.

Because the study used a single self-reported Likert-scale questionnaire, common method variance (CMV) was assessed. Harman's single-factor test was conducted using unrotated exploratory factor analysis. In the pilot sample (*n* = 50), the first factor accounted for 29.4% of the total variance, indicating no serious single-factor dominance. In the main sample (*n* = 864), the first factor explained 32.7% of the total variance, which was below the commonly used threshold, suggesting that CMV was unlikely to pose a serious threat to the main findings.

In addition, because the sample covered students from different academic disciplines and year levels, subgroup robustness was considered in the analysis plan. To strengthen the interpretability and generalizability of the findings across major subgroups, the study further examined whether the measurement structure and key relationships were reasonably stable across discipline- and year-based categories. This step was included to reduce the risk that the reported structural paths reflected subgroup-specific measurement patterns rather than the broader institutional–psychological mechanism proposed in the model. Furthermore, given the on-site field administration approach, the interpretation of results emphasizes structural relationships within the sampled population while acknowledging potential self-selection bias in external generalization.

## 4 Results

### 4.1 Descriptive statistics and demographic analysis

The demographics of the sample is contained in Table 1. The sample size was 864 students of the university, and the ratio of males to females was relatively even (45.0:55.0). The sampling was fairly balanced across academic years, consisting of freshmen, sophist, juniors, and seniors in 27.0, 22.5, 25.5, and 25.1 percent of the sample, respectively. Academically, students of medicine, natural sciences, and social sciences constituted 31.7, 34.7, and 33.6 percent of the sample, respectively. Most of the students (69.6) studied in the public universities and only 30.4 in the private institutions. On the family income aspect, 25.6% claimed to be low income earners, 50.7% were medium income earners, and 23.7% were high income earners. These demographic spreads show that the sample represents the main population of the university students in the Shanxi Province well.

**Table 1:** Demographic characteristics of the sample (*N* = 864).

Demographic variable	Category	Frequency	Percentage
Gender	Male	389	45.0
	Female	475	55.0
Academic year	Freshman	233	27.0
	Sophomore	194	22.5
	Junior	220	25.5
	Senior	217	25.1
Academic discipline	Medicine	274	31.7
	Natural sciences	300	34.7
	Social sciences	290	33.6
University type	Public	601	69.6
	Private	263	30.4
Family income	Low	221	25.6
	Medium	438	50.7
	High	205	23.7

Table 2 demonstrates descriptive statistics data on the primary variables. The standard deviations were 0.33–1.15 with mean scores of all constructs between 1.34 and 2.73. All the skewness and kurtosis levels were within acceptable levels (skewness less than 1.0 and kurtosis less than 3.0) implying that the data was more or less normal, which was suitable in further structural equation modeling.

**Table 2:** Descriptive statistics for main variables (*N* = 864).

Variable	Mean	SD	Skewness	Kurtosis
University Leadership (UL)	2.73	1.15	0.18	−0.85
Sustainability-oriented education	2.71	1.11	0.22	−0.72
Environmental Values (EV)	2.11	0.85	0.31	−0.58
Environmental Beliefs (EB)	1.47	0.48	0.42	−0.41
Personal Norms (PN)	1.34	0.33	0.35	−0.29
Pro-environmental Behavioral Intention (PEBI)	2.09	0.83	0.39	−0.51

It should be noted that the mean scores for Environmental Beliefs (EB = 1.47) and Personal Norms (PN = 1.34) were relatively low, with limited dispersion, especially for PN (SD = 0.33). To verify that this pattern was not caused by data-processing errors, the coding direction of all items was rechecked prior to analysis, and no reverse-coding inconsistency was identified in the final dataset. Therefore, the low means are interpreted as reflecting a low baseline level of environmental moral obligation and belief activation in the sampled student population, rather than a scoring artifact. Although the absolute levels were low, the constructs still showed acceptable psychometric quality and meaningful covariance with other variables, supporting their continued inclusion in the SEM analysis. Nevertheless, the restricted variance in these constructs should be considered when interpreting the magnitude of the structural path coefficients.

### 4.2 Measurement model: reliability and validity assessment

Confirmatory factor analysis (CFA) was used to measure the validation of the model. Cronbachs alpha and Composite Reliability (CR) measures of reliability were used (Hair et al., 2022). As presented in Table 3, all the constructs had excellent internal consistency with Cronbach's alpha ranging between 0.88 and 0.93 (all above the 0.70 level), and CR values ranging between 0.91 and 0.95 (all above the 0.70 level).

**Table 3:** Reliability and validity of the measurement model.

Construct	Items	Factor loadings	Cronbach's α	CR	AVE
UL	5	0.78–0.88	0.912	0.930	0.725
SE	5	0.80–0.86	0.898	0.921	0.698
EV	4	0.79–0.85	0.885	0.910	0.715
EB	5	0.78–0.89	0.925	0.940	0.758
PN	4	0.81–0.87	0.905	0.925	0.755
PEBI	5	0.83–0.88	0.931	0.945	0.775

Factor loading was tested, as well as AVE (Hair et al., 2019), to test convergent validity. Standardized factor loadings of all factors were between 0.78 and 0.89, which is higher than the stipulated 0.70. The AVE is 0.698 to 0.775, whereas it is above 0.50. Such findings validate the assumption that the measurement items can be used to measure its constructs.

Fornell-Larcker criterion was used to test discriminant validity; a comparison of square root of construct AVE and correlations with other constructs was undertaken (Fornell and Larcker, 1981). Table 4 indicates that all the diagonal values (square roots of AVE) were higher than the off-diagonal correlations provided in the rows and columns of the table, and that each of the constructs is independent enough of the others.

**Table 4:** Discriminant validity (Fornell-Larcker criterion).

Construct	UL	SE	EV	EB	PN	PEBI
UL	0.851					
SE	0.512	**0.835**				
EV	0.445	0.489	**0.845**			
EB	0.298	0.325	0.412	**0.871**		
PN	0.215	0.241	0.285	0.398	**0.869**	
PEBI	0.298	0.356	0.521	0.512	0.621	**0.880**

Diagonal values in bold represent the square root of AVE for each construct.

### 4.3 Structural model and hypothesis testing

The structural model demonstrated an acceptable overall fit to the data (χ^2^/*df* = 2.38, *p* < 0.001; CFI = 0.943; TLI = 0.932; RMSEA = 0.051; SRMR = 0.067), indicating that the hypothesized relationships adequately reproduced the observed covariance structure and providing a sound basis for interpreting the structural paths (Hu and Bentler, 1999; Schermelleh-Engel et al., 2003). As reported in Table 5, 11 out of 13 direct-effect hypotheses were supported, suggesting that the proposed framework is largely consistent with the data while also revealing clear heterogeneity in the effectiveness of different institutional levers.

**Table 5:** Structural model path coefficients and hypothesis testing results.

Hypothesis	Path	Standardized β	*t-value*	*p-value*	Result
H1a	UL → PEBI	0.068	1.287	0.198ns	Not Supported
H1b	UL → EV	0.312	5.876	^***^	Supported
H1c	UL → EB	0.198	3.654	^***^	Supported
H1d	UL → PN	0.087	1.542	0.123ns	Not Supported
H2a	SE → EV	0.245	4.892	^***^	Supported
H2b	SE → EB	0.287	5.543	^***^	Supported
H2c	SE → PN	0.156	2.876	^**^	Supported
H2d	SE → PEBI	0.218	4.321	^***^	Supported
H3	EV → PEBI	0.195	4.567	^***^	Supported
H4	EB → PEBI	0.201	4.892	^***^	Supported
H5	PN → PEBI	0.287	6.543	^***^	Supported
H6	EV → EB	0.456	9.876	^***^	Supported
H7	EB → PN	0.398	8.234	^***^	Supported

*** p < 0.001,

** p < 0.01, ^*^p < 0.05, ns, not significant.

UL, University Leadership; SE, Sustainability-oriented Education; EV, Environmental Values; EB, Environmental Beliefs; PN, Personal Norms; PEBI, Pro-environmental Behavioral Intention.

Regarding institutional predictors, university leadership (UL) showed a differentiated pattern of effects. UL did not exert a statistically significant direct influence on pro-environmental behavioral intention (PEBI) (H1a: β = 0.068, *t* = 1.287, *p* = 0.198) and also failed to significantly predict personal norms (PN) (H1d: β = 0.087, *t* = 1.542, *p* = 0.123). In contrast, UL significantly and positively predicted environmental values (EV) (H1b: β = 0.312, *t* = 5.876, *p* < 0.001) and environmental beliefs (EB) (H1c: β = 0.198, *t* = 3.654, *p* < 0.001), indicating that leadership effects were more pronounced at the level of upstream cognitive orientations than at the level of the most proximal motivational determinant of intention. Sustainability-oriented education, by comparison, exhibited robust, and comprehensive effects across outcomes. SE significantly predicted EV (H2a: β = 0.245, *t* = 4.892, *p* < 0.001), EB (H2b: β = 0.287, *t* = 5.543, *p* < 0.001), PN (H2c: β = 0.156, *t* = 2.876, *p* < 0.01), and PEBI (H2d: β = 0.218, *t* = 4.321, *p* < 0.001), with the direct path to PEBI reaching a moderate magnitude, implying that educational interventions contribute to intention formation both directly and through downstream psychological processes.

The Value–Belief–Norm (VBN) chain was strongly supported. EV significantly predicted EB (H6: β = 0.456, *t* = 9.876, *p* < 0.001), and EB significantly predicted PN (H7: β = 0.398, *t* = 8.234, *p* < 0.001), providing empirical confirmation of the sequential structure from values to beliefs to moral obligation. Moreover, EV, EB, and PN each had significant positive direct effects on PEBI (H3: β = 0.195, *t* = 4.567, *p* < 0.001; H4: β = 0.201, *t* = 4.892, *p* < 0.001; H5: β = 0.287, *t* = 6.543, *p* < 0.001). Regarding the strength of the effects, PN proved to be the best direct predictor of PEBI, which is in line with the assumption that moral obligation is the most proximate cause of pro-environmental intention. Combined with Tables 4, 5 suggests a solid psychological chain with institutionalized differences in effects: sustainability-oriented education has important effects on all levels of the VBN pathway, as well as on any stage of intention formation, but the influence of university leadership is much stronger on the formation of values and beliefs at the upper levels, but not on the translation of this into personal norms and intention.

### 4.4 Mediation effects analysis

To test mediation (H8a–H10c), data was tested to see the indirect effects through bootstrapping with (5,000 resamples and 95% confidence interval) (Preacher and Hayes, 2008). Table 6 shows the results.

**Table 6:** Mediation effects analysis.

Mediation hypothesis	Mediation path	Indirect effect	95% CI	Significance
H8a	UL → EV → PEBI	0.061	[−0.008, 0.142]	Not Significant
H8b	SE → EV → PEBI	0.048	[0.012, 0.098]	Significant
H8c	UL → EV → EB	0.142	[0.089, 0.201]	Significant
H8d	SE → EV → EB	0.112	[0.065, 0.167]	Significant
H9a	UL → EB → PEBI	0.040	[−0.005, 0.089]	Not Significant
H9b	SE → EB → PEBI	0.058	[0.021, 0.101]	Significant
H9c	UL → EB → PN	0.079	[0.034, 0.132]	Significant
H9d	SE → EB → PN	0.114	[0.067, 0.168]	Significant
H9e	EV → EB → PN	0.182	[0.128, 0.242]	Significant
H10a	UL → PN → PEBI	0.025	[−0.012, 0.068]	Not Significant
H10b	SE → PN → PEBI	0.045	[0.015, 0.082]	Significant
H10c	EB → PN → PEBI	0.114	[0.072, 0.161]	Significant

Significant mediation effects are indicated when the 95% confidence interval does not include zero.

UL, University Leadership; SE, Sustainability-oriented Education; EV, Environmental Values; EB, Environmental Beliefs; PN, Personal Norms; PEBI, Pro-environmental Behavioral Intention.

Table 7 summarizes the proposed framework, and overall, the empirical evidence is able to support the proposed framework with most of the hypothesized relationships established. Particularly, direct-effect hypotheses are highly supported, which shows that the integrated model contains both institutional factors and the basic VBN psychological pathway to elucidate the energy saving intentions held by students. The support rate of mediation hypothesis is also high, which implies that indirect transmission is one of the key mechanisms in which institutional factors do influence intention. It is noteworthy that the unsupported hypotheses are focused on UL-related associations with PEBI, both directly and indirectly with the key mediators, and SE-related mechanisms are always supported. This evidence on preferred vs. rejected hypotheses underlines the key conclusion of Section 4.4 namely that explanatory power is mainly provided by sustainability-oriented education and the VBN chain, whereas leadership influences appear to be relatively constrained and less predictable to convert institutional signals into behavioral intention.

**Table 7:** Summary of hypothesis testing results.

Category	Total hypotheses	Supported	Not supported	Support rate
Direct effects (H1a–H7)	13	10	3	76.9%
Mediation effects (H8a–H10c)	12	9	3	75.0%
Total	25	19	6	76.0%

The fact that the overall support rate of 76.0% in Table 7 indicates that there is a high level of validity in the proposed theoretical model, however, the existence of significant boundary conditions and limitations to the institutional effects should be investigated further. The tendency of non-supported hypotheses implies that the effect of institutional factors (leadership and education) is not unanimous on all psychological outcomes, and their impact on behavior is mostly indirect and implemented by activating beliefs and personal norms.

## 5 Discussion

### 5.1 Research findings

One of the most notable findings of this study is that university leadership did not show a significant direct effect on students' pro-environmental behavioral intention. This result challenges a common assumption in organizational and higher education literature that university leadership can directly translate into intended student outcomes. A more plausible explanation lies in the structural nature of leadership in higher education and the psychological distance between leadership actions and students' everyday energy-use behavior. At the macro-institutional level, university leadership is typically expressed through strategic plans, policy statements, and resource allocation decisions. These actions are often abstract and relatively distant from students' day-to-day experiences on campus. Even when sustainability is declared an institutional priority, students may not perceive such declarations as personally relevant to their own energy consumption behavior. This pattern is consistent with the implementation gap discussed in organizational studies, namely the gap between what leaders formally promote and what organizational members actually experience in practice. In the context of campus sustainability, students may recognize that their university promotes “green” initiatives, but this recognition may not automatically translate into an internalized sense of responsibility for energy conservation. This interpretation is consistent with Maqsoom et al. (2025), who found that leadership effects on pro-environmental behavior were primarily transmitted through intermediate psychological mechanisms rather than direct influence. Similarly, Ahsan et al. (2024) reported that authentic leadership tends to affect sustainable behavioral intention by activating values and cognition rather than by directly prompting behavior.

The non-significant direct leadership effect (H1a) may also reflect a perceived mismatch between symbolic sustainability messaging and students' everyday observations of institutional practice. Students in higher education settings are increasingly capable of evaluating institutional rhetoric critically, and leadership messages may lose persuasive power when they are not accompanied by visible and tangible changes in campus operations. One possible interpretation is that some students may perceive sustainability initiatives as largely symbolic or performative, which may reduce the persuasive power of leadership signals. However, this study did not directly measure students' perceptions of symbolic communication or institutional credibility, so this explanation should be treated as an interpretive possibility rather than a confirmed mechanism. This interpretation helps explain why leadership messaging alone may fail to generate behavioral intention. In this sense, the H1a result suggests that leadership influence on student energy-saving intention may require not only policy-level positioning, but also concrete institutional practices that students can directly observe and experience. It is also important to note that the UL construct in this study reflects students' perceived university leadership (i.e., the leadership signals students can directly observe in campus life), rather than an objective measure of top-level university governance capacity. Therefore, the non-significant direct effect should be interpreted as a limitation in the behavioral influence of perceived leadership cues at the student level, rather than as evidence that university leadership is unimportant in a broader administrative sense.

A further explanation for the non-significant H1a result is the temporal and spatial distance between leadership decisions and student behavior. University leadership typically operates at a strategic level, often over long planning cycles and campus-wide agendas, whereas student energy-saving behavior occurs in immediate and localized settings such as dormitories, classrooms, and daily routines. This mismatch in temporal and spatial scale may weaken the direct influence of leadership on behavioral intention. In contrast, more proximal factors may play a more immediate role in shaping students' energy-saving intentions, such as whether energy-saving reminders are present in dormitory environments, whether peers model conservation behavior, or whether there are concrete incentives linked to daily practice. This interpretation is consistent with the proximity principle in environmental psychology, which suggests that people are more responsive to environmental cues and social influences that are physically and temporally close to the decision context (Shooshtarian et al., 2025).

The findings also suggest that university leadership is less effective in activating personal norms, which represent an internalized sense of moral obligation. Personal norms are typically formed through direct experience, moral reflection, and close interpersonal interaction rather than through remote institutional messaging. This helps explain why leadership may influence students' environmental beliefs (as reflected in the significance of H1c) but still fail to trigger a stronger sense of personal moral responsibility toward energy conservation (H1d). In other words, recognizing that environmental problems are serious and that one bears some responsibility for them is not necessarily equivalent to internalizing a personal duty to act. The latter generally requires a deeper process of psychological internalization. Moral psychology research similarly indicates that personal norms are shaped through value meaning, socialization processes in immediate social environments, and direct experience with the consequences of one's behavior (Yaban and Gaschler, 2025). From this perspective, leadership communication may support awareness, but it may be less effective in producing the internal moral commitment represented by personal norms.

Relatedly, students may interpret leadership-led sustainability efforts as externally driven rather than internally meaningful, which may further limit the formation of personal norms. If sustainability messages are perceived primarily as institutional requirements, image-oriented communication, or top-down expectations, students may be less likely to internalize them as part of their own moral framework. This interpretation is consistent with reactance-based explanations in prior research, which suggest that individuals may reduce acceptance of messages they perceive as externally controlling (Zhou et al., 2025). In this context, leadership communication may still raise awareness, but it may be less effective in fostering self-endorsed moral obligation.

The results further imply that personal norms may be shaped more effectively through peer-related and experience-based processes than through formal institutional authority. Emerging research on social influence in environmental behavior suggests that the perceived values and behaviors of one's close social group can have a strong effect on the development of moral responsibility (Gelfand et al., 2024). In university settings, roommates, classmates, and friendship groups are part of students' daily social environment and may therefore exert more immediate influence on whether energy conservation becomes morally salient. This pattern suggests that interventions relying solely on top-down leadership communication may be less effective than those that also create peer-supported social conditions in which energy-saving behavior is normalized.

The non-significant indirect effect H8a (University Leadership → Environmental Values → PEBI: IE = 0.061, 95% CI = −0.008 to 0.142, ns) highlights an important limitation in the leadership-to-behavior pathway. Although leadership significantly influenced environmental values (H1b), these values did not significantly transmit leadership influence to behavioral intention. This result suggests that value activation alone may be insufficient to convert institutional influence into specific energy-saving intention. Environmental values are broad guiding principles regarding what is important (e.g., protection of nature), whereas energy-saving intention refers to concrete and specific behavioral plans. The translation from general values to specific behavior often depends on additional cognitive and motivational processes. This interpretation is consistent with research on the value-action gap, which shows that people may endorse environmental values without consistently translating those values into everyday pro-environmental action (Trujillo and Luchs, 2025). In the present context, the values activated by leadership may operate at a general attitudinal level but may not be sufficiently internalized or behaviorally specific to support intention formation.

A related explanation is that leadership may activate environmental values at a broad normative level, while the behavioral outcome in this study is highly specific. Students may agree in principle that environmental protection is important, yet this broad endorsement may not readily translate into intention to perform specific energy-saving actions. This is also consistent with the specificity principle in attitude–behavior research, which suggests that prediction improves when the level of specificity between predictor and outcome is aligned (Burgos-Espinoza et al., 2025). The non-significance of H8a therefore suggests that leadership-led sustainability efforts may need to move beyond broad value messaging and provide stronger links to concrete energy-saving responsibilities and practices.

Although university leadership significantly influenced environmental beliefs (H1c), the indirect path from leadership through environmental beliefs to behavioral intention was not significant (H9a). This pattern suggests that increasing environmental beliefs alone may not be enough to generate behavioral intention unless students also perceive clear, feasible, and personally relevant action routes. Students may believe that energy conservation is important and that individuals bear responsibility for energy-related problems, but such beliefs may not translate into intention when students are uncertain about the practical impact of their own actions or lack concrete strategies for energy saving. This interpretation is consistent with environmental psychology research emphasizing the role of efficacy-related cognition in converting concern into intention (Song et al., 2024).

The contrast between leadership and sustainability-oriented education is also informative here. Environmental beliefs activated through leadership messaging may remain relatively general, whereas beliefs developed through educational processes are more likely to be linked to practical understanding and actionable knowledge. Educational interventions can more directly connect environmental problems with feasible daily energy-saving strategies, thereby strengthening the translation from belief to intention. From this perspective, the non-significance of H9a suggests that leadership-based influence through beliefs may remain limited unless it is reinforced by educational content that provides clear behavioral guidance.

Taken together, the results indicate that university leadership appears less effective in activating the personal norm pathway, and this has important implications for understanding institutional influence on student energy-saving intention. In the VBN framework, personal norms function as the most proximate moral driver of behavioral intention. However, leadership operates through institutional authority and hierarchical communication, which may be less conducive to the kind of internalization required for personal norm formation. In contrast, educational processes are more likely to support internalization because they provide knowledge, interpretation, and reflection opportunities that students can integrate into their own moral reasoning. This helps explain why sustainability-oriented education showed a stronger and more consistent role in activating personal norms than leadership did.

The same logic also helps explain why peer and experience-based processes remain important. Personal norms are often strengthened through socialization, role modeling, and direct experience. In university life, peers are socially and spatially closer to students than institutional administrators, which may make peer influence more salient in shaping moral obligation related to everyday energy use (Zhou and Zheng, 2024). This pattern suggests that institutional efforts to promote energy conservation may benefit from combining formal sustainability initiatives with peer-based engagement mechanisms and student-led sustainability activities. This interpretation is also consistent with the relatively low mean scores observed for environmental beliefs and personal norms in the descriptive results. These low baseline levels suggest that, in the sampled student population, environmental concern may not yet have been strongly internalized as personal responsibility or moral obligation in everyday campus energy-use contexts. This pattern further helps explain why educational intervention appeared more effective than leadership cues in activating downstream normative commitment.

In contrast to the limitations observed for university leadership, sustainability-oriented education showed strong and consistent effects across the model. Education significantly influenced environmental values, environmental beliefs, personal norms, and behavioral intention, both directly and through mediation pathways. This pattern underscores the central role of education in shaping pro-environmental intention among university students and suggests that educational interventions may function as the most effective institutional channel for activating the VBN process.

The stronger effects of sustainability-oriented education can be understood through several complementary mechanisms. Educational interventions provide action-oriented knowledge that helps students connect environmental issues with specific behavioral responses. Compared with abstract leadership messaging, educational content can be made more personally relevant and easier to internalize. In addition, education is often delivered through repeated and context-rich formats—such as classroom teaching, workshops, and campus-based information sessions—which may strengthen reinforcement over time. Educational settings also allow instructors to function as credible knowledge sources and supportive guides, making students more receptive to sustainability-related meaning and practice. These features help explain why sustainability-oriented education appears more effective than leadership communication in activating multiple layers of the psychological pathway from values and beliefs to norms and behavioral intention.

Finally, the findings provide substantial support for the VBN causal chain in the context of university students' energy-saving intention. Personal norms emerged as the strongest direct predictor of pro-environmental behavioral intention, which is consistent with the core VBN proposition that moral obligation is the most proximal driver of intention. In addition, the significant mediation role of environmental beliefs within the VBN pathway (e.g., H9e) further highlights the importance of belief formation as an intermediate mechanism linking values to moral obligation and intention. However, the results also show that environmental values and environmental beliefs retained significant direct effects on pro-environmental behavioral intention. This means that the present findings are better interpreted as a partial-mediation pattern rather than a strict full-mediation version of VBN. In the HEI energy-saving context, upstream psychological factors appear to influence intention both through the normative pathway and through direct value- and belief-based routes. Overall, the results suggest that effective campus energy-saving interventions should not rely on information provision alone, but should also support the internalization of responsibility and the translation of environmental cognition into concrete daily behavioral commitment.

### 5.2 Theoretical contributions

The findings of the present study contribute to a more context-sensitive understanding of how institutional influence operates in university students' pro-environmental intention formation. The non-significant direct effect of university leadership, together with its significant effects on environmental values and environmental beliefs, suggests that institutional influence may be transmitted more effectively through intermediate psychological mechanisms than through a direct behavioral pathway (Maqsoom et al., 2025). The strong and stable effects of sustainability-oriented education observed in the present model further indicate that educational support may function as a more behaviorally proximal form of institutional influence in higher education settings (Qi et al., 2025). This interpretation is reinforced by evidence showing that environmental education can directly promote concrete pro-environmental behavior among college students, rather than merely serving as a general background condition (Luo et al., 2025). It has also been indicated that university-based contextual conditions can shape students' pro-environmental behavior by influencing the psychological and situational conditions surrounding action (Fu et al., 2025). Taken together, these comparisons suggest that institutional influence in higher education should be understood as a layered process in which different institutional conditions shape different stages of students' pro-environmental intention formation.

A further theoretical contribution lies in the extension of the Value–Belief–Norm framework in the higher education energy-saving context. Traditional applications of VBN have primarily emphasized the internal progression from values to beliefs, from beliefs to personal norms, and from personal norms to behavioral intention. The present findings indicate that this internal sequence can be more fully understood when institutional support is incorporated as an upstream contextual condition. This position is supported by research showing that educational conditions in universities can generate measurable effects on students' environmentally relevant behavior, thereby shaping the motivational foundation of pro-environmental action (Luo et al., 2025). It has also been shown that contextual influences within universities can contribute to the emergence of students' pro-environmental behavior, which supports the theoretical placement of institutional support ahead of the internal VBN process (Fu et al., 2025). On this basis, the present study further suggests that institutional support should not be treated as a single undifferentiated construct. Sustainability-oriented education was linked more strongly and more consistently to environmental values, environmental beliefs, personal norms, and pro-environmental behavioral intention. By contrast, university leadership was linked more clearly to upstream cognitive components, especially values and beliefs, but less strongly to personal norms and downstream behavioral intention. This differentiated pattern enriches the application of VBN in higher education by showing that distinct institutional mechanisms may activate different stages of the psychological pathway.

A third theoretical implication concerns the form of mediation within the VBN pathway. Although the sequential VBN structure was supported, significant direct effects were also retained by environmental values and environmental beliefs on pro-environmental behavioral intention. This pattern suggests that the present findings are better interpreted as reflecting partial mediation rather than a strict full-mediation version of VBN. This interpretation is consistent with research indicating that personal norms remain a particularly strong predictor of university students' energy-saving intention in campus-related settings (Ling and Ling, 2025). It is also consistent with evidence from student residential energy contexts, where energy-saving intention has been shaped by highly proximal and context-sensitive psychological processes (Hou and Law, 2024). These comparisons suggest that specific daily energy-saving intentions among university students may not be fully explained by a purely linear progression from distal cognition to action. In the higher education energy-saving context, upstream psychological factors appear to influence intention both indirectly through personal norms and directly through value- and belief-based motivational routes. In this respect, the present study refines the application of VBN in higher education by showing that moral obligation remains the strongest proximal driver, while distal value and belief components may continue to retain independent explanatory power under institutional sustainability conditions.

### 5.3 Practical implications

The findings suggest that sustainability-oriented education should be treated as a central component of university energy-conservation strategies. Given its consistent effects across environmental values, environmental beliefs, personal norms, and pro-environmental behavioral intention, universities are advised to strengthen the integration of sustainability-related content into formal curricula, campus campaigns, and practice-based learning activities. Educational interventions appear especially valuable when they connect environmental issues with concrete, student-relevant actions.

The results also indicate that university leadership remains important, but its role may be more effective as an enabling and coordinating force than as a direct driver of student behavioral intention. In this study, leadership showed stronger influence on upstream psychological variables than on direct intention formation. Therefore, university leadership may be better positioned to provide policy support, allocate resources, and create institutional conditions that reinforce educational interventions, rather than relying mainly on top-down messaging to produce behavioral change.

A further implication is that universities should pay greater attention to the development of students' personal norms, because personal norms emerged as the strongest direct predictor of pro-environmental behavioral intention in the VBN chain. Since leadership signals alone appear less effective in activating personal moral obligation, universities may benefit from designing peer-based and student-centered sustainability initiatives. Examples include dormitory energy-saving campaigns led by student teams, student sustainability clubs, and peer-led participation mechanisms that make energy conservation more socially visible and morally meaningful in daily campus life.

The findings also suggest that sustainability interventions should not stop at raising general environmental awareness. While environmental values and beliefs are important, the results indicate that abstract environmental concern alone may be insufficient to generate strong behavioral intention. Universities should therefore complement awareness-building with practical guidance, such as clear energy-saving routines, actionable behavioral tips, and context-specific strategies that help students translate environmental concern into concrete energy-conservation intentions.

Finally, the results imply that universities should avoid relying primarily on symbolic or low-visibility sustainability communication. When sustainability efforts are communicated mainly at a rhetorical level, students may perceive them as distant from everyday campus life, which can weaken behavioral influence. Universities may therefore improve effectiveness by linking sustainability priorities to visible institutional actions and to educational content that helps students understand both the practical relevance of energy conservation and their own role in achieving it. This combination of visible institutional commitment and actionable educational support is likely to provide a stronger foundation for fostering pro-environmental intentions among students.

## 6 Conclusion

The present study contributes to the literature on pro-environmental behavior among university students by offering a more nuanced explanation of how institutional support shapes pro-environmental behavioral intention in higher education energy-saving contexts. The findings indicate that university leadership does not exert a significant direct effect on students' pro-environmental behavioral intention and does not significantly activate personal norms, which represent the most immediate and powerful motivational basis of intention. By contrast, sustainability-oriented education shows strong and significant effects across environmental values, environmental beliefs, personal norms, and pro-environmental behavioral intention, both directly and through multiple mediation pathways. These results suggest that educational support functions as a more immediate and effective institutional mechanism for promoting student energy-saving intention than leadership signals alone. Strong support was also found for the Value–Belief–Norm (VBN) framework, with personal norms emerging as the strongest direct predictor of pro-environmental behavioral intention. At the same time, the differentiated pattern of mediation indicates important boundary conditions: institutional support appears to be most effective in activating environmental beliefs, moderately effective in shaping environmental values, and comparatively less effective in fostering the deeper sense of moral obligation reflected in personal norms.

In practical terms, the findings suggest that universities seeking to strengthen students' energy-saving intention should adopt a multi-layered intervention strategy. Sustainability-oriented education should be treated as a core institutional mechanism, because it provides students with concrete knowledge, interpretive guidance, and action-oriented understanding of energy conservation. Universities are therefore encouraged to integrate sustainability-related content more systematically into curricula, co-curricular activities, and campus campaigns. At the same time, university leadership remains important, but its role appears to be more effective as an enabling and coordinating force than as a direct driver of student behavioral intention. Leadership efforts should therefore focus on providing policy support, allocating resources, and creating institutional conditions that strengthen student-centered and education-led sustainability action. The findings also indicate that peer-based engagement may be especially important for activating personal norms. Universities may therefore benefit from investing in student-led sustainability projects, dormitory-based energy-saving campaigns, and peer-supported participation mechanisms that make energy conservation more socially visible and morally meaningful in everyday campus life. By combining direct educational strategies with leadership support for student-driven change, universities may more effectively foster sustained pro-environmental behavior among students.

Despite these contributions, several limitations should be acknowledged. First, the study relied on a single self-reported questionnaire, which may be subject to social desirability bias, perceptual bias, and the broader limitation that reported intention does not always translate into actual energy-saving behavior. Although common method variance was assessed and did not appear to pose a serious threat, the use of self-reported data still limits the extent to which the findings can be interpreted as reflecting actual behavioral practice. Second, the sample was drawn from full-time undergraduate students in Shanxi Province, China, and the findings should therefore be generalized with caution to other regional, cultural, or institutional settings. Differences in policy environments, campus governance structures, and sustainability cultures across universities or countries may lead to different patterns of institutional and psychological influence.

Future research could address these limitations in several ways. Behavioral or longitudinal data could be incorporated to examine whether reported intention is translated into sustained energy-saving practice over time. Broader sampling across multiple provinces, institutional types, or national contexts would also help determine whether the present institutional support–VBN relationships remain stable across different higher education environments. In addition, the integrated institutional support–VBN framework could be extended by incorporating perspectives such as self-determination theory to examine the role of perceived autonomy and intrinsic motivation in mediating institutional effects. Further work could also explore moderating mechanisms such as institutional credibility, peer influence strength, and students' environmental identity in order to clarify the conditions under which institutional and psychological factors most effectively promote sustained pro-environmental behavior.

## Data Availability

The raw data supporting the conclusions of this article will be made available by the authors, without undue reservation.

## Appendix

**Table A1:** Measurement items.

Construct	Code	Statement
University Leadership (UL)	UL1	My department heads are committed to promoting energy conservation and environmental protection on campus.
	UL2	Department heads in my university encourage students to participate in energy-saving and other environmental activities.
	UL3	Students in my department are encouraged by the department heads and teachers to volunteer for energy conservation initiatives.
	UL4	I believe that the department heads of my university will not compromise on sustainability and energy-saving principles.
	UL5	I believe that my department heads uphold high standards and values regarding sustainability and energy conservation.
Sustainability-Oriented Education (SE)	SE1	Sustainability-oriented education is provided at all levels within the university.
	SE2	Sustainability-related courses and activities are accessible to all students in the university.
	SE3	There are effective institutional policies ensuring access to sustainability-oriented education in the university.
	SE4	The participation rate in sustainability-oriented education programs is high in the university.
	SE5	The right to participate in sustainability-oriented education is guaranteed to all students in the university.
Environmental Values (EV)	EV1	Preventing pollution and conserving natural resources by reducing unnecessary energy use on campus is important to me.
	EV2	Respecting the earth and protecting other species by lowering energy-related environmental pressure is important to me.
	EV3	Protecting the environment and preserving nature through daily energy conservation is important to me.
	EV4	I value fair and responsible use of shared campus energy resources.
Environmental Beliefs (EB)	EB1	I believe that excessive energy use on campus contributes to environmental problems.
	EB2	I believe that reducing unnecessary energy use on campus can help protect the environment.
	EB3	I believe my personal energy-saving behavior can contribute to reducing energy waste on campus.
	EB4	I believe students share responsibility for improving energy conservation on campus.
	EB5	I believe universities should take stronger action to support energy conservation and environmental protection.
Personal Norms (PN)	PN1	I feel morally accountable to reduce energy waste and environmental problems on campus.
	PN2	People like me should make efforts to reduce unnecessary energy use and support a more sustainable campus environment.
	PN3	I feel personally obliged to support energy-saving efforts that help improve the campus environment.
	PN4	I believe it is morally important to contribute to campus energy saving and environmental well-being, regardless of what others do.
Pro-environmental Behavioral Intention (PEBI)	PEBI1	I will try to reduce electricity use whenever I am on campus.
	PEBI2	I intend to switch off lights and air-conditioning when they are not in use.
	PEBI3	I plan to open windows for ventilation before turning on the air-conditioning.
	PEBI4	I will make sure to switch off electronic and electrical equipment after each class.
	PEBI5	I intend to participate in energy-saving or awareness activities on campus.
